# A Narrative Review of the Evidence for Variations in Serum 25-Hydroxyvitamin D Concentration Thresholds for Optimal Health

**DOI:** 10.3390/nu14030639

**Published:** 2022-02-02

**Authors:** William B. Grant, Fatme Al Anouti, Barbara J. Boucher, Erdinç Dursun, Duygu Gezen-Ak, Edward B. Jude, Tatiana Karonova, Pawel Pludowski

**Affiliations:** 1 Sunlight, Nutrition, and Health Research Center, P.O. Box 641603, San Francisco, CA 94164-1603, USA; 2 Department of Health Sciences, College of Natural and Health Sciences, Zayed University, Abu Dhabi 144534, United Arab Emirates; fatme.alanouti@zu.ac.ae; 3 The Blizard Institute, Barts & The London School of Medicine & Dentistry, Queen Mary University of London, London E12AT, UK; bboucher@doctors.org.uk; 4 Department of Neuroscience, Institute of Neurological Sciences, Istanbul University-Cerrahpasa, Istanbul 34098, Turkey; erdincdu@gmail.com (E.D.); duygugezenak@gmail.com (D.G.-A.); 5 Tameside and Glossop Integrated Care NHS Foundation Trust, Fountain Street, Ashton-under-Lyne OL6 9RW, UK; Edward.jude@tgh.nhs.uk; 6 The University of Manchester, Oxford Road, Manchester M13 9PL, UK; 7 Manchester Metropolitan University, All Saints Building, Manchester M15 6BH, UK; 8 Clinical Endocrinology Laboratory, Department of Endocrinology, Almazov National Medical Research Centre, 194021 Saint-Petersburg, Russia; karonova@mail.ru; 9 Department of Biochemistry, Radioimmunology and Experimental Medicine, The Children’s Memorial Health Institute, 04730 Warsaw, Poland; p.pludowski@ipczd.pl

**Keywords:** Alzheimer’s disease, cancer, cardiovascular disease, COVID-19, diabetes, hypertension, Mendelian randomization, vitamin D, 25-hydroxyvitamin D

## Abstract

Vitamin D_3_ has many important health benefits. Unfortunately, these benefits are not widely known among health care personnel and the general public. As a result, most of the world’s population has serum 25-hydroxyvitamin D (25(OH)D) concentrations far below optimal values. This narrative review examines the evidence for the major causes of death including cardiovascular disease, hypertension, cancer, type 2 diabetes mellitus, and COVID-19 with regard to sub-optimal 25(OH)D concentrations. Evidence for the beneficial effects comes from a variety of approaches including ecological and observational studies, studies of mechanisms, and Mendelian randomization studies. Although randomized controlled trials (RCTs) are generally considered the strongest form of evidence for pharmaceutical drugs, the study designs and the conduct of RCTs performed for vitamin D have mostly been flawed for the following reasons: they have been based on vitamin D dose rather than on baseline and achieved 25(OH)D concentrations; they have involved participants with 25(OH)D concentrations above the population mean; they have given low vitamin D doses; and they have permitted other sources of vitamin D. Thus, the strongest evidence generally comes from the other types of studies. The general finding is that optimal 25(OH)D concentrations to support health and wellbeing are above 30 ng/mL (75 nmol/L) for cardiovascular disease and all-cause mortality rate, whereas the thresholds for several other outcomes appear to range up to 40 or 50 ng/mL. The most efficient way to achieve these concentrations is through vitamin D supplementation. Although additional studies are warranted, raising serum 25(OH)D concentrations to optimal concentrations will result in a significant reduction in preventable illness and death.

## 1. Introduction

Vitamin D deficiency is the most common nutritional deficiency in the world, although vitamin D is one of the most well understood compounds. It reduces risks of many adverse health outcomes through both genetic and non-genetic mechanisms; it is readily available from supplements, is safe, and inexpensive. There are over 94,000 publications on vitamin D listed on pubmed.gov as of 20 December 2021. Despite all this, vitamin D deficiency (25-hydroxyvitamin D (25(OH)D) concentration <20 ng/mL) is very common, affecting about half of the world’s population [1], whereas the higher concentrations necessary for optimal non-skeletal health (25(OH)D >30 ng/mL) are not so common. The reason for this is that the beneficial effects of vitamin D for non-skeletal disorders have only received widespread research attention since 2000. Furthermore, methods for studying the many health effects of vitamin D have been evolving and researchers have had to identify their various strengths and limitations. Another factor is that conventional medicine in most countries generally focuses on treatment rather than on the prevention of disease.

This is a narrative review of what is known about the role of vitamin D supplementation and how it raises serum 25(OH)D concentration and influences health outcomes as well as achieves maximum reductions of mortality rates in developed countries from the commonest fatal diseases (e.g., cancer, cardiovascular disease including hypertension, COVID-19 and diabetes mellitus type 2). In addition, an overview is included of the types of studies used to evaluate the effects of vitamin D supplementation, and of the different serum 25(OH)D concentrations required to achieve various health outcomes, with outlines of the strengths and limitations of the various types of studies. Circulating 25(OH)D is important as the precursor of the most active vitamin D metabolite, calcitriol (1,25-dihydroxyvitamin D) formed by the 1-alpha hydroxylation of 25(OH)D in the kidneys and many other immune and metabolically active tissues as needed.

The approach taken in this report is to base recommendations on the strongest evidence available, generally from observational studies that are supported by other types of studies. The strengths and weaknesses of the various types of studies, ranging from ecological studies to meta-analyses of randomized controlled trials (RCTs) and Mendelian randomization (MR) studies are discussed at the end of the discussion. Although meta-analyses are often considered to be stronger evidence than individual studies, the fact that many studies have flaws in their design, conduct, or analysis, means that some individual studies are better suited for making recommendations than many meta-analyses. 

The background for the present report is provided by data on mortality rates for major causes of death in 2016 by the World Health Organization (see Table 1 for Germany, Japan, Saudi Arabia, and the U.S.). This study will concentrate on the diseases with the highest prevalence or mortality rates. 

It is apparent from the data in Table 1 that there are large differences in mortality rates by country and sex. National diet plays an important role in health outcomes. Spending on health care can affect outcomes. Obesity increases adverse health outcomes. Smoking has many adverse health effects. Males tend to smoke more than females, and this may explain why mortality rates for males are higher than for females. Although we do not discuss the effect of factors other than vitamin D and solar UVB in this study, we acknowledge that raising serum 25(OH)D concentrations may not have the same effects in all participants.

## 2. Results

### 2.1. Cardiovascular Disease

Robert Scragg was the first to propose that the adequate provision of vitamin D might reduce the risk of cardiovascular disease (CVD) since incidence and mortality rates were highest in winter when solar UVB doses and serum 25(OH)D concentrations were lowest [2]. Markedly higher rates of CVD risk factors were found in winter than in summer in 24 population-based studies in 14 countries [3]. The risk factors considered included BMI, waist circumference systolic and diastolic blood pressure, total, high- and low-density lipoprotein cholesterol, triglycerides, and glucose levels. 

Thomas Wang and colleagues published the first observational study of CVD risk with respect to serum 25(OH)D concentration in a prospective study of participants in the Framingham Offspring Study in 2008 [4]. Several vitamin D-related mechanisms appear to have protective roles for CVD including the known inhibition of vascular smooth muscle proliferation, the suppression of vascular calcification, the reduction of inflammation through the regulation of cytokines, and the regulation of blood volume and systemic vascular resistance through renin gene suppression [5].

Observational studies have shown that serum 25(OH)D concentrations correlate inversely with CVD incidence and mortality rates and also with data for coronary or ischemic heart disease, congestive heart failure and stroke. Acute cardiovascular events are commonly precipitated by plaque disruption following local inflammation and the release of destructive MMPs, especially MMP9, from invading foam macrophages. Inflammation is suppressed by vitamin D and non-skeletal MMP2/9 production is suppressed by vitamin D; circulating MMP2/9 concentrations relate inversely to serum 25(OH)D and can be suppressed by modest supplementation [6].

In clinical trials, vitamin D supplementation has been found to reduce the serum levels of total cholesterol (TC), low-density lipoprotein cholesterol (LDL-C), and triglycerides (TG) but not high-density lipoprotein cholesterol (HDL-C) [7]. 

A study of 20,025 patients in the U.S. Veterans Health Administration system followed for 20 years found that those with no previous myocardial infarction (MI) who had basal 25(OH)D concentrations ≤20 ng/mL and raised their concentrations to >20 ng/mL significantly reduced their risk of MI [8]. For those achieving 25(OH)D concentrations ≥30 ng/mL vs. 21 to 29 ng/mL, the hazard ratio (HR) for MI was 0.65 (95% CI, 0.49 to 0.85, *p* = 0.002), whereas for those who achieved 25(OH)D concentrations ≥30 ng/mL vs. ≤20 ng/mL, the HR for MI was 0.73 (95% CI, 0.55 to 0.96, *p* = 0.02). 

Similarly, a study based on UK Biobank participants who had CVD followed for a median of 11.7 years showed a linear inverse association of CVD mortality (*P*-non-linearity = 0.07) with 25(OH)D, the adjusted HR decreasing from 1.31 (95% CI, 1.14 to 1.43) at 4 ng/mL to 0.83 (95% CI, 0.77 to 0.88) at 15 ng/mL and then (linearly) to 0.37 (95% CI, 0.22 to 0.62) at 60 ng/mL [9].

Support for the role of vitamin D in reducing the risk of CVD also comes from observational associations and MR analyses of four population-based cohort studies (UK Biobank, EPIC-CVD and two Copenhagen population-based studies) comprising 386,406 middle-aged individuals of European ancestries [10] followed after enrollment, with blood drawn from between 9 to 21 years. In the observational part of this study, coronary heart disease events were significantly increased below 12 ng/mL, stroke events below 20 ng/mL, and the CVD mortality rate below 25 ng/mL. In that MR analysis, a 4 ng/mL increase in genetically predicted 25(OH)D was associated with an OR for stroke of 0.85 (95% CI, 0.70 to 1.02, *p* = 0.09) and for coronary heart disease of 0.89 (95% CI, 0.76 to 1.04, *p* = 0.14). However, these values probably underestimate the true value due to changes in 25(OH)D over time. An analysis of HR vs. the follow-up period for observational studies of all-cause mortality rate for an 8 ng/mL difference in 25(OH)D concentration over six to fourteen years found a linear increase in HR from 0.81 (95% CI, 0.67 to 1.02) at six years to 0.96 (95% CI, 0.9 to 1.01) at fourteen years [11].

Subsequently, an MR study based on UK Biobank data for 20,805 incident CVD events amongst 267,980 subjects with both serum 25(OH)D concentration data and phenotypic 25(OH)D analyses [12] reported that each 4 ng/mL increase in phenotypic 25(OH)D was associated with a 1.6% lower risk of a CVD event (OR = 0.98 (95% CI, 0.98 to 0.99, *p* = 0.0001)) for 25(OH)D concentrations of up to 50 ng/mL. However, the genetic (MR) analysis using data for 35 vitamin D-related single nucleotide polymorphisms (SNPs) found in the 295,788 participants including 44,519 CVD cases, revealed that there was an L-shaped relationship, with 11% (95% CI, 1.05 to 1.18) higher odds for CVD events at 10 ng/mL than at 20 ng/mL; moreover, for 25(OH)D concentrations around 4 ng/mL, the odds were +2.3 (95% CI, 1.6 to 3.7) using the non-linear analytical approach of Staley and Burgess [13] on the 25(OH)D data after the data had been divided into 100 equal strata, with a plateau above ~20 ng/mL.

### 2.2. Hypertension

Good evidence now shows that vitamin D status affects the risk and prevalence of hypertension. For example, a meta-analysis of seven out of eight prospective cohort studies with 283,537 participants found the risk of developing hypertension for those in the upper third vs. lower third of 25(OH)D concentrations was reduced by 30% (RR = 0.70 (95% CI, 0.58 to 0.86)) [14] with an overall RR for incident hypertension for every 10 ng/mL increment in baseline 25(OH)D concentration of 0.88 (0.81, 0.97) using dose-response analysis.

An MR study on 142,225 participants of European descent from several countries with measurements of systolic and diastolic blood pressure and genetic analyses [15] found four variants of genes affecting 25(OH)D synthesis from vitamin D (*CYP2R1*) through involvement in the synthesis of 7-dehydrocholesterol, which is converted to vitamin D_3_ in the skin via UVB irradiance followed by a thermal process involving *DHCR7*, the gene providing instructions for making an enzyme called 7-dehydrcholesterol reductase. In their meta-analysis, a ’synthesis score’ was associated with a reduced risk of hypertension (OR per allele, 0.98, 0.96–0.99; *p* = 0.001). Each 10% increase in genetically determined 25(OH)D concentration was associated with a reduction of −0.29 mm Hg in diastolic blood pressure (95% CI, −0.52 to −0.07; *p* = 0.01), of −0.37 mmHg in systolic blood pressure (95% CI, −0.73 to 0.003; *p* = 0.052), and an 8% decreased risk of hypertension (OR 0.92, 95% CI, 0.87–0.97; *p* = 0.002). Although these differences in blood pressure were small, that was likely due to the limitations of the MR analyses which had few alleles of genes affecting serum 25(OH)D concentrations to consider. 

An observational study of community-based participants taking ~4000 IU/day of vitamin D_3_ to achieve 25(OH)D concentrations >40 ng/mL reported significant reductions in blood pressure and hypertension prevalence after a year [16]. At baseline, 592 participants (7.3%) were hypertensive. At follow-up (12 ± 3 months), 71% of them were no longer hypertensive. The mean 25(OH)D concentration for those hypertensive participants increased from 33 ± 16 ng/mL to 45 ± 14 ng/mL with increased vitamin D supplementation (from ~2000 IU/day to ~6000 IU/day). For those not taking hypotensive medication, systolic BP decreased by 18 ± 19 mmHg and diastolic BP fell by 12 ± 12 mmHg, whereas in those taking hypotensive medication after joining the program, systolic BP decreased by 14 ± 21 mmHg and diastolic BP decreased by 12 ± 12 mmHg, whereas there were no changes in blood pressure in the normo-tensive control participants.

### 2.3. Cancer 

It was first proposed in 1980 that sunlight reduced the risk of colon cancer with vitamin D production being the likely reason [17]. Since then, numerous ecological studies have reported that solar UVB dose indices correlate inversely with incidence and/or mortality rates for nearly 20 types of cancer [18,19,20]. The best ecological studies are those from single mid-latitude countries where variations in solar UVB doses tend to be large [21,22], whereas variations in other risk-modifying factors (diet, skin pigmentation, dress, obesity, smoking, alcohol consumption, etc.) are often small or can be accounted for [19]. Thus, ecological studies provide a case for examining whether better vitamin D provision reduces cancer incidence or cancer mortality.

Observational studies have shown associations between cancer risk and solar UV radiation. A meta-analysis of 14 studies observed reduced breast cancer rates for those spending ≥1 h/day in sunlight during summer months over their lifetime or adulthood compared to <1 h/day (RR = 0.84; 95% CI: 0.77, 0.91) [23]. Another meta-analysis of six studies between 2005 and 2020 found an inverse correlation between exposure to solar UV radiation and breast cancer risk (RR: 0.70, 95% CI: 0.65, 0.75), [24]. In total, 17 case-control studies and 9 cohort studies, including 216,285 non-Hodgkin’s lymphoma (NHL) and 23,017 Hodgkin’s lymphoma (HL) patients, were included in the final analysis. Personal sunlight exposure was significantly associated with reduced risks of HL (OR = 0.77; 95% CI 0.68–0.87) and of all types of NHL (OR = 0.81; 95% CI 0.71–0.92) other than T-cell lymphoma [25]. Furthermore, no mechanism other than the production of vitamin D has been suggested to explain the protective effects of solar UV against cancer.

There are many observational studies of cancer incidence with serum 25(OH)D concentrations. The meta-analyses of observational studies are shown in Table 2. The reason case-control studies report greater risk reductions is probably the long follow-up times of cohort studies providing baseline 25(OH)D concentrations which become less well correlated with cancer outcomes over time [26]. Although serum 25(OH)D concentration can be reduced by acute inflammatory illness [27], cancer does not appear to have this effect.

Although the data in Table 2 demonstrate that serum 25(OH)D concentration is commonly inversely correlated with cancer incidence, they do not provide data on the relationship of 25(OH)D concentration to cancer incidence, although other studies do so. For breast cancer, two studies can be used. One is a meta-analysis of 36 case-control and four cohort studies [29] where a spline fit to the data reveals a 60% (95% CI, 45% to 70%) reduction in risk with 25(OH)D concentrations from 4 ng/mL up to 40 ng/mL and an 80% reduction with 25(OH)D concentrations of up to 80 ng/mL. However, the primary data source for 25(OH)D concentrations of >40 ng/mL is from an observational study based on data from women enrolled in vitamin D RCTs who took vitamin D supplements (1000 or 2000 IU/day) or a placebo [31,32] or were enrolled in a volunteer cohort who took doses of their choice and had serum 25(OH)D measured half-yearly [33]. This pooled cohort included 5028 women out of whom 77 developed incident breast cancer. There was an 82% lower incidence rate for 25(OH)D concentrations >60 ng/mL vs. <20 ng/mL (*p* = 0.006) and, importantly, the slope of that relationship was similar for subjects with values both below and above 40 ng/mL.

For colorectal cancer, a 2019 meta-analysis [34] using data from case-control studies of women with 25(OH)D concentrations between <15 ng/mL and >29 ng/mL found that the relative risk (RR) per 10 ng/mL increase in 25(OH)D was 0.81 (95% CI, 0.75 to 0.87), whereas for men with 25(OH)D concentrations between <16 and >30 ng/mL, the RR was 0.93 (95% CI, 0.86 to 1.00). Thus, for rises in 25(OH)D ranging from ~10 ng/mL to ~35 ng/mL, the RR was 0.59 for women and 0.83 for men. The increase in RR seen with 25(OH)D concentrations of >100 ng/mL vs. <40 ng/mL is likely due to participants starting to supplement shortly prior to enrolling in cohort studies [35] and should not necessarily be taken to indicate that higher 25(OH)D concentrations reverse the beneficial effects found at lower 25(OH)D concentrations, especially since the ecological study of cancer mortality rates in the U.S. between 1950 and 1994 showed that UVB dose–cancer mortality rate curves plateaued at the highest UVB exposures [36]. 

The effect of vitamin D in reducing cancer mortality rates appears to be more significant than for reducing cancer incidence. For example, the VITamin D and OmegA-3 TriaL (VITAL) study conducted by Harvard University [37] did not find a significant reduction in all-cancer incidence with vitamin D supplementation vs. placebo for the entire set of participants but did find a significantly reduced risk of mortality when the first two years of data were omitted, HR = 0.75 (0.59–0.96). Furthermore, another analysis of vitamin D RCTs also found a greater reduction for mortality rates than for incidence rates (Table 3). However, meta-analyses of observational studies found significant reduction for both all-cancer incidence and mortality rates (Table 4).

Additional support for the effects of vitamin D provision on cancer risks is that many mechanisms exist through which vitamin D reduces cancer risks, including effects on cells that are anti-proliferative, pro-differentiating, anti-inflammatory, immunomodulatory, anti-angiogenic around tumors, and anti-metastatic [40,41,42,43]. The active vitamin D metabolite (1α,25-dihydroxyvitamin D_3_ or calcitriol) inhibits proliferation and promotes the epithelial differentiation of human colon carcinoma cell lines that express the vitamin D receptor (VDR) via the regulation of a large number of genes [44]. Other RCTs reported that the higher the vitamin D supplement given (up to 10,000 IU/day), the more genes whose expression is changed [45,46]. No adverse effects of supplementation with 10,000 IU/day were found in these studies. Thus, the known mechanisms support the finding that a higher 25(OH)D threshold can reduce cancer risks and cancer mortality.

Most vitamin D–cancer RCTs seem to have failed because they were designed using guidelines developed for assessing pharmaceutical drugs, not nutrients [47,48]. As a result, baseline 25(OH)D concentrations were often too high and vitamin D doses were generally too low to correct deficiency. Furthermore, changes in serum 25(OH)D concentrations differ individually in response to supplementation [49], and other sources of vitamin D, including unknown intakes from self-supplementation in both treatment and placebo arms, cannot be allowed for. 

A meta-analysis of vitamin D RCTs regarding breast cancer incidence included eight trials comprising 72,275 participants with median follow-up periods ranging from 1 to 11.9 years. The doses were 400 to 1100 IU/day in four trials, 2000 IU/day in two trials, and 100,000 IU/month in two trials. This study found RRs of 1.04 (95% CI 0.85–1.29, *p* = 0.68) for vitamin D supplementation (6 trials, 33,472 participants, 246 events), and 0.99 (95% CI 0.91–1.07, *p* = 0.73) for vitamin D plus calcium (4 trials, 41,957 participants, 2195 events) [50]. The doses were too low and/or too infrequent in the trials of monthly dosing since the half-life of 25(OH)D is about 20 days; thus, it is not surprising that the RR findings were not significant.

The inspection of VITAL study results, where the mean baseline and achieved 25(OH)D concentrations for those with measured values were 27.8 and 39.7 ng/mL for males and 31.7 and 43.6 ng/mL for females, and using a vitamin D_3_ dose of 2000 IU/d, the HR for overall cancer incidence was 0.96 (95% CI, 0.88 to 1.06). However, for black participants, where the mean reported baseline and achieved 25(OH)D concentrations were 25.0 and 39.7 ng/mL, respectively, the HR was 0.77 (95% CI, 0.59 to 1.01) and for participants with a BMI <25 kg/m^2^ with a mean reported baseline and achieved 25(OH)D concentrations of 33.3 and 45.9 ng/mL, respectively, the HR for cancer incidence was 0.76 (95% CI, 0.63 to 0.90) [37]. 

In conclusion, the evidence that higher vitamin D status reduces the risk of cancer incidence includes:

1—Single-country ecological studies finding that about 20 types of cancer have incidence and mortality rates inversely correlated with various indices of solar UVB doses.

2—Observational studies reporting that several types of cancer have incidence rates inversely correlated with serum 25(OH)D in case-control or cohort observational studies.

3—An observational study using individual participant data for women taking vitamin D or placebo in two RCTs or taking vitamin D in a volunteer cohort, and with some subjects achieving 25(OH)Ds > 60 ng/mL, achieving a significant reduction in breast cancer incidence.

4—Mechanisms that explain how vitamin D reduces the risk of cancer incidence, progression, and metastasis. 

5—No mechanisms are yet known that might explain how non-vitamin D mechanisms associated with UVB exposure could reduce the risk of cancer.

### 2.4. Type 2 Diabetes Mellitus 

Type 2 diabetes mellitus (T2DM) is a condition in which there is too much glucose circulating in the blood because of long-standing increases in insulin resistance leading to the eventual deficiency in insulin responsiveness to hyperglycaemia [51,52]. Although T2DM is generally associated with obesity and ‘cafeteria’ diets, there is now reasonably strong evidence that risk is inversely associated with serum 25(OH)D concentration and that correcting vitamin D deficiency over time can reduce T2DM risks. 

The mechanisms by which vitamin D reduces the risk of T2DM include β-cell insulin release through a rise in intracellular calcium concentration [53] and by stimulating insulin synthesis [54], which was reported in 1980 [55] (see review in [56]). Vitamin D reduces insulin resistance by reducing oxidative stress and inflammation and reducing both hepatic lipogenesis and hepatic glucose release through metformin-like effects [57,58] and by promoting increased metabolic efficiency in skeletal muscle [59,60]. Thus, it is not surprising that obesity, which is marked by increased systemic inflammation as well as lower 25(OH)D concentrations [61], and a lack of physical activity are major risk factors for T2DM [62].

Prospective observational studies support a role for vitamin D in reducing T2DM risks. A meta-analysis of 16 prospective studies published in 2013 found an OR = 1.50 (95% CI, 1.33 to 1.67) for the incidence of T2DM for low vs. high 25(OH)D status in each study [63]. Large prospective studies have shown that lower 25(OH)D status is a risk factor for metabolic syndrome [64] and for T2DM [65].

Higher 25(OH)D concentrations are associated with lower mortality rates for adults with diabetes prospectively. A study was reported on 6329 adults with diabetes from the Third National Health and Nutrition Examination Survey (NHANES III) and NHANES 2001–2014 followed up through 31 December 2015 [66]. In 55,126 person-years of follow-up, 2056 deaths were documented (605 from CVD and 309 from cancer). Compared with participants with 25(OH)D concentrations <25 nmol/L, the multivariate-adjusted HRs and 95% CI for participants with 25(OH)D concentrations >75 nmol/L were 0.59 (95% CI, 0.43, 0.83) for all-cause mortality (*p*_trend_ = 0.003), 0.50 (95% CI, 0.29, 0.86) for CVD mortality (*p*_trend_ = 0.02), and 0.49 (95% CI, 0.23, 1.04) for cancer mortality (*p*_trend_ = 0.12).

A meta-analysis of 24 RCTs (*n* = 1528 individuals with T2DM) found significant reductions in glycosylated hemoglobin (HbA1c) (mean difference: −0.30%; 95% CI: −0.45 to −0.15, *p* < 0.001), serum fasting plasma glucose (FPG) (mean difference: −4.9 mg/dL (−0.27 mmol/L); 95% CI: −8.1 to −1.6 (−0.45 to −0.09 mmol/L), *p* = 0.003), and the homeostatic model assessment of insulin resistance (HOMA-IR) (mean difference: −0.66; 95% CI: −1.06 to −0.26, *p* = 0.001) in diabetic patients [67] where a mean increase in 25(OH)D concentration in those RCTs was 17 ± 2 ng/mL, suggesting that a minimum dose of 4000 IU/day of vitamin D is advisable for improving insulin sensitivity and glycemic control in T2DM patients.

It seems that vitamin D supplementation in doses of 4000 IU/day or higher improved the manifestation of diabetic complications. An example would be the study conducted in St. Petersburg, Russia that included 62 T2DM patients with diabetic polyneuropathy [68]. The intake of 40,000 IU/week of vitamin D for 24 weeks was associated with an increased 25(OH)D concentration from 16 to 72 ng/mL and a reduction in neurological deficit and pain severity, as well as improved interleukin profiles and markers for microcirculation. No overall changes were detected in T2DM patients who received 5000 IU/week of vitamin D. 

The Vitamin D and Type 2 Diabetes (D2d) Study conducted by Tufts University is the largest RCT to examine the effect of vitamin D supplementation on the risk of T2DM [69]. A total of 2423 prediabetic participants were enrolled and half were given 4000 IU/day of vitamin D_3_, whereas the other half were given a placebo during a mean 2.5-year follow-up period. The HR for progression to T2DM for the treatment arm compared to the control arm was 0.88 (95% CI, 0.75 to 1.04, *p* = 0.12). Two subgroups had a significantly reduced risk when comparing treatment to control groups, those with BMI <30 kg/m^2^ (HR = 0.71 (95% CI, 0.53 to 0.95)) and those not taking calcium (HR = 0.81 (95% CI, 0.66 to 0.98)). However, it was a secondary analysis related to 25(OH)D concentration maintained through the RCT that provided strong support for vitamin D supplementation reducing the progression from pre-diabetes to T2DM [70]. Over the range from 20–30 ng/mL to >50 ng/mL, for each increase in 25(OH)D by 10 ng/mL, those in the treatment arm had an HR of 0.75 (95% CI, 0.68 to 0.82) for conversion to T2DM. Therefore, secondary analysis based on serum 25(OH)D concentration is clearly an appropriate way to analyze RCT results since vitamin D, being a nutrient provided from several sources, has non-linear effects in contrast to a ‘medication’ [47,48].

### 2.5. COVID-19

The world is in the midst of the COVID-19 pandemic with nearly 5.6 million deaths reported to date (21 January 2022) (https://www.worldometers.info/coronavirus/ (accessed on 5 January 2022)). On 2 April 2020, it was suggested that vitamin D could reduce the risk of COVID-19 through several mechanisms including inducing the secretion of cathelicidins and defensins known to reduce viral replication rates and reduce the production of pro-inflammatory cytokines aggravating the inflammation that injures the lungs, leading to pneumonia. Vitamin D also increases the secretion of anti-inflammatory cytokines, overall reducing the risk of highly dangerous cytokine storms [71]. The important modulatory effect of vitamin D on immune-related genes through binding to VDR implicates a potential role in clearing SARS-CoV-2 infections [72].

The rationale for that suggestion included the observation that case fatality rates in the U.S. during the 1918–1919 influenza pandemic were mainly due to the development of pneumonia and were lowest in communities with the highest solar UVB doses [73]. In addition, a meta-analysis of RCTs had shown a reduction in acute respiratory tract infections with supplementation in vitamin D deficiency cases using analyses of individual participant data [74]. 

Observational studies suggest that vitamin D reduces SARS-CoV-2 infection risk. A study of >190,000 patients who had SARS-CoV-2-positive tests in the U.S. between 9 March and 19 June 2020 and had their serum 25(OH)D concentration measured during the previous twelve months (by Quest Diagnostics, Secaucus, NJ) [75] showed the following results by race/ethnicity, after adjusting for seasonal differences: black non-Hispanics, Hispanics, and white non-Hispanics had ~19%, 16% and 9% rates for a 25(OH)D concentration <20 ng/mL, respectively, and rates of 11%, 10%, and 5%, respectively for 25(OH)D values of ~55 ng/mL. These values represent reductions by about 40% for all three races/ethnicities. The higher SARS-CoV-2 positivity rates for black American non-Hispanics and Hispanics was mostly likely due to their being in lower socioeconomic strata and more likely to be unable to socially isolate or to work from home than white non-Hispanics as well as being more likely to have been aggravated by their lower vitamin D status, itself known to worsen with lower SE status as well as being increased in those with darker skin [76].

There have been many observational studies. Some were retrospective studies where 25(OH)D concentrations for those becoming ill and being diagnosed with COVID-19 were obtained from measurements made before diagnosis (seasonally adjusted). Examples include those from Israel [77] and Chicago, IL, USA [78,79]. However, the majority of such observational investigations were based on 25(OH)D concentration at the time of diagnosis upon hospital admission [80].

The most recent meta-analysis of COVID-19 risk in relation to serum 25(OH)D concentrations was published on 11 December 2021 [81]. It included results from 76 observational studies. Vitamin D deficiency/insufficiency increased the odds of developing COVID-19 (OR 1.46, 95% CI 1.28–1.65, *p* < 0.0001), developing severe disease (OR 1.90, 95% CI 1.52–2.38, *p* < 0.0001) and death (OR 2.07, 95% CI 1.28–3.35, *p* = 0.003). A major concern is that having COVID-19 must itself lower 25(OH)D concentration as is usual in severe infection, as shown experimentally [27]. This concern will eventually be clarified by examining data for COVID-19 outcomes by pre-illness and/or pre-pandemic 25(OH)D concentration in comparison with those found at the time of diagnosis. In one meta-analysis of vitamin D deficiency/insufficiency and risk of COVID-19 involving 19 studies, the OR was 1.46 (95% CI, 1.28 to 1.65). Of the 19 studies, ten had 25(OH)D concentrations measured prior to COVID-19 diagnosis, of which three were 10–15 years before the pandemic. The ORs for those three were near 1.00 and non-significant, as expected for such a long lag time. Examining the five studies with values measured in the previous year (omitting one that is a preprint), the ORs for four of them were above the mean value and one was very near the meta-analysis value, 1.46. Thus, on the basis of that analysis, there does not seem to be a significant difference between risks relating to low 25(OH)D values whether measured before or at the time of COVID-19 diagnosis.

The most recent article on vitamin D and the risk of COVID-19 hospitalization and mortality was published on 1 January 2022 [82]. It presented an analysis of 4599 veteran patients receiving care in the US Department of Veterans Affairs health care facilities who tested positive for SARS-CoV-2 during the period from 20 February to 8 November 2020 and who had serum 25(OH)D concentration data from the previous 15 to 90 days on file. Twenty one percent of the patients were hospitalized and 7.4% died within 60 days of their index SARS-CoV-2 test. Hospitalization rates decreased from 25% at 15 ng/mL to 18% at 60 ng/mL (adjusted relative risk = 1.29 (95% CI, 1.06 to 1.57), whereas morality rates decreased from 11% for vitamin D levels of 15 ng/mL to 6% at 60 ng/mL (adjusted relative risk = 1.82 (1.27 to 2.63)).

Results of a trial conducted in Turkey involving 132 COVID-19 patients with a baseline 25(OH)D concentration <30 ng/mL, of whom 80 were treated with high-dose vitamin D3 to achieve a 25(OH)D concentration >30 ng/mL, was reported recently [83]. Vitamin D_3_ doses ranged from 224,000 to 500,000 IU over periods from three to 14 days. The mean 25(OH)D for the treated patients reached only 31 ± 12 ng/mL on day 7 and 35 ± 11 ng/mL on day 14. The mortality rate was 11.2% (97 out of 867) in the whole cohort. The mortality rate for patients who had comorbidities but received vitamin D treatment was 5.5% (9 out of 162). Having vitamin D treatment decreased the 14-day mortality rate significantly (OR for survival: 2.14, 95%CI: 1.06 to 4.33, *p* = 0.03).

Calcifediol (25(OH)D_3_) is being used in Spain to treat COVID-19 patients, its advantage being that it increases serum 25(OH)D concentrations within hours and much faster than intact vitamin D_3_. The first study of the calcifediol treatment of COVID-19 patients was conducted in Cordoba, Spain [84] in 76 consecutive patients admitted to a university hospital with clinically diagnosed COVID-19 who all received standard care with hydroxychloroquine plus azithromycin for patients with pneumonia (a broad-spectrum antibiotic). Fifty of these patients were ‘randomized’ to receive oral calcifediol on the day of admission at 0.532 mg followed by 0.266 mg on days 3 and 7 and then weekly until discharge or intensive care unit (ICU) admission. Calcifediol is ~×3 times more effective in raising serum 25(OH)D concentration (after adjusting for weight) than vitamin D_3_. The only significant difference in the prognostic factors for COVID-19 at baseline was previous high blood pressure (at 24% in the treatment group and 58% in the control group; *p* = 0.002). Only one of the treated patients but 13 of the untreated patients required admission to the ICU (*p* < 0.001). There was no death among treated patients, but two untreated patients requiring care in the ICU died.

Another Spanish report summarized the results of treating 537 COVID-19 patients admitted to any of the five hospitals in southern Spain between 5 February and 5 May 2020 with calcifediol (25(OH)D_3_) [85], excluding the 76 patients already mentioned [84]. In that study, 79 patients were treated and 458 were not treated with calcifediol. The untreated patients had significantly higher rates of CURB-65 ≥3 (21 vs. 8%) and ARDS (25 vs. 10%), higher CRP (130 ± 100 vs. 100 ± 80 units) and blood urea nitrogen (22 ± 19 vs. 16 ± 15 units) values together with lower oxygen saturation at admission (93 ± 6% vs. 95 ± 4%). However, those differences were not mortality rate determinants. The crude OR for death in patients treated with calcifediol vs. those not so treated was 0.22 (95% CI, 0.08 to 0.61, *p* < 0.01) and was 0.16 (95% CI, 0.03 to 0.80) after adjustment for all other risk factors. On the other hand, higher age, ARDS, CURB-65 ≥3, cerebrovascular disease, COPD, cancer, and ratio of neutrophils to lymphocytes were significantly associated with increased mortality. Other studies have shown that low serum 25(OH)D concentration is a stronger marker of adverse outcomes than those other factors. For example, in an observational study in Iran involving 442 patients in general wards and 66 patients in the ICU, of whom 55 died, only age (*p* < 0.001, albumin (*p* < 0.001), calcium, (*p* = 0.002) and serum 25(OH)D (*p* = 0.047)) were significantly associated with mortality in multivariate analysis, whereas BMI, diabetes mellitus, hypertension, IHD, creatinine, and phosphorus were not [86]. 

Another review used meta-analyses on COVID-19 hospitalized patient outcomes [87]. Using two studies that gave vitamin D_3_ and two giving calcifediol, the OR for intensive care unit admission was 0.27 (95% CI, 0.09 to 0.76); on the other hand, based on four studies giving vitamin D_3_ and one giving calcifediol, the OR for needing mechanical ventilation was 0.34 (95% CI, 0.16 to 0.72) with these treatments. Overall, from those eight studies plus one more, the OR for mortality for any form of vitamin D/calcifediol treatment was 0.37 (95% CI, 0.21 to 0.66). 

An observational study in Barcelona compared the incidence of COVID-19 for patients with respect to serum 25(OH)D concentration and whether they were being treated with vitamin D_3_ or calcifediol [88]. For those being treated with vitamin D_3_ and achieving >30 ng/mL, the multivariate HR compared to untreated controls with <20 ng/mL for SARS-CoV-2 infection was 0.66 (95% CI, 0.57 to 0.77), 0.72 (0.52 to 1.00) for severe COVID-19, and 0.66 (0.46 to 0.93) for COVID-19 mortality. Similarly, for those being treated with calcifediol, the multivariate HR for SARS-CoV-2 infection was 0.69 (95% CI, 0.61 to 0.79), 0.61 (0.46 to 0.81) for severe COVID-19, and 0.56 (0.42 to 0.76) for COVID-19 mortality. The small differences between vitamin D and calcifediol treatment may be due to different effective vitamin D doses and achieved 25(OH)D concentrations. Thus, there is accumulating evidence that higher serum 25(OH)D concentrations protect against COVID-19, but more research is warranted.

### 2.6. Alzheimer’s Disease

Alzheimer’s disease (AD) is a neurodegenerative disease of the brain, generally with increased beta-amyloid plaque and tau protein deposition. The mechanisms whereby vitamin D reduces the risk of AD include preventing amyloid development and clearing it from the brain [89,90,91]. Most of the studies on the effects of vitamin D on the risk of AD are observational. One from France followed 916 participants over 65 years old for 12 years [92]. A total of 117 dementia cases developed, of which 124 were AD. The adjusted HR for AD compared to 25(OH)D concentrations >20 ng/mL was 2.17 (95% CI, 1.37 to 5.68) for those with a 25(OH)D concentration between 12 and 25 ng/mL and 2.85 (95% CI, 1.36 to 5.97) for those with a 25(OH)D concentration <12 ng/mL.

A meta-analysis of six observational studies found the HR for AD for a 10 ng/mL increase in 25(OH)D concentration of 0.83 (95% CI, 0.68 to 0.96) [93]. Another meta-analysis based on nine observational studies found that for 25(OH)D concentrations <20 ng/mL, HR = 1.34 (95% CI, 1.13 to 1.60) [94]. A recent MR analysis based on data from the IGAP and the UK Biobank found that genetically increased 25(OH)D concentrations were significantly associated with reduced risks of AD [95].

### 2.7. All-Cause Mortality 

Based on the effect of vitamin D on the major causes of death in developed countries, it would be expected that there would be an inverse relationship between serum 25(OH)D concentrations and subsequent all-cause mortality rate and this has been found using meta-analyses; one conducted in 2012 used 14 prospective studies with 62,548 individuals found an RR of 0.69 (95% CI, 0.60 to 0.78) for 25(OH)D = 31 ng/mL vs. 11 ng/mL, with no further decreases in mortality rates above 35 ng/mL [96]. Another meta-analysis of 32 studies found that 25(OH)D values >30 ng/mL vs. <10 ng/mL had an RR for survival of 1.9 (95% CI, 1.6 to 2.2) [97]. Another study from a European consortium of 26,916 individuals found that mortality rates increased with a lower individual participant standardized 25(OH)D concentration in a cubic spline model adjusted for age, sex, and BMI at baseline visit, compared to 30–40 ng/mL, HR = 1.06 (0.96 to 1.15) for 25(OH)D concentrations reduced to 20–30 ng/mL, 1.14 (1.03–1.24) for 25(OH)D concentrations of 16–20 ng/mL, and reaching 1.29 (1.17–1.41) for 25(OH)D concentrations of 12–16 ng/mL, and 1.72 (1.53–1.90) for 25(OH)D values <12 ng/mL [98]. In the 20-year Veterans Health Administration study regarding myocardial infarction discussed above [8], patients achieving 25(OH)D concentrations of 20–30 ng/mL vs. <20 ng/mL had an all-cause mortality HR of 0.59 (95% CI, 0.54 to 0.63), whereas in those achieving values >30 ng/mL, the mortality HR was not further reduced at 0.61 (95% CI, 0.56 to 0.67).

A meta-analysis of 52 trials with 75,454 participants in vitamin D RCTs found that supplementation was not associated with any changes in all-cause mortality rate (RR = 0.98 (95% CI, 0.95 to 1.02)) [99]. However, vitamin D supplementation did significantly reduce cancer mortality rate (RR = 0.84 (95% CI, 0.74 to 0.95)) and subgroup analyses showed all-cause mortality was significantly lower in trials giving vitamin D_3_ supplementation vs. placebo (RR = 0.95 (95% CI, 0.91 to 1.00)) than in trials with vitamin D_2_ supplementation vs. placebo (RR = 1.03 (95% CI, 0.98 to 1.09)). One problem with those meta-analyses was that they did not have individual participant data for analysis, as was used for the acute respiratory tract infection meta-analysis already referred to [74]. Another concern is that trials with large cohorts used in those meta-analyses were made variously between 1996 to 2018, when 25(OH)D assays used were changing over [100,101], which inevitably adds a degree of error to the reported meta-analyses.

## 3. Discussion

A summary of the findings reported in this review is given in Table 5. The optimal 25(OH)D concentration thresholds for these various outcomes range from 25 ng/mL to 60 ng/mL. All of these concentrations are higher than the 20 ng/mL recommended by the Institute of Medicine based on its interpretation of requirements for bone health [102]. They are in general agreement with the Endocrine Society’s recommendation of >30 ng/mL [103], based on a more careful interpretation of a study of 25(OH)D concentrations and bone mineralization [104]. They are also consistent with a recommendation of 30–50 ng/mL in 2018 for the pleiotropic (non-skeletal) effects of vitamin D [105]. 

The 25(OH)D concentration range of 30–40 ng/mL could generally be met by the supplementation of 2000 to 4000 IU/day, which was reported as safe for all by the Institute of Medicine [102]. Achieving concentrations above 40 ng/mL could take higher doses. The Institute of Medicine noted that they did not have evidence that taking up to 10,000 IU/day of vitamin D had any adverse effects, but set the upper tolerable level at 4000 IU/day out of a concern for safety. The UK NIH also agrees that 4000 IU/day is safe (https://www.nhs.uk/conditions/vitamins-and-minerals/vitamin-d/ accessed on 4 January 2021).

It has been shown experimentally that humans can produce between 10,000 and 25,000 IU of vitamin D through whole-body exposure to one minimal erythemal dose of simulated sunlight, i.e., one instance of mid-day sun exposure without burning [107]. Thus, doses to those levels should be considered inherently safe. Recent articles have reported the safety results for high-dose vitamin D supplementation. One was a community-based, open-access vitamin D supplementation program involving 3882 participants conducted in Canada between 2013 and 2015 [108]. Participants took up to 15,000 IU/day of vitamin D_3_ for between 6 and 18 months. The goal of the study was to determine vitamin D doses required to achieve a 25(OH)D concentration >40 ng/mL. It was found that participants with a normal BMI had to take at least 6000 IU/day of vitamin D, whereas overweight and obese participants had to take 7000 IU/day and 8000 IU/day, respectively. Serum 25(OH)D concentrations of up to 120 ng/mL were achieved without the perturbation of calcium homeostasis or toxicity.

Another study involved 777 long-term hospitalized patients taking 5000 to 50,000 IU/day of vitamin D_3_ [109]. Subsets of those taking 5000 IU/d achieved mean 25(OH)D concentrations of 65 ± 20 ng/mL after 12 months, whereas those taking 10,000 IU/day achieved 100 ± 20 ng/mL after 12 months. No patients who achieved 25(OH)D concentrations of 40–155 ng/mL developed hypercalcemia, nephrolithiais (kidney stones), or any other symptoms of vitamin D toxicity as the result of vitamin D supplementation. 

Hypersensitivity to vitamin D can develop in people with sarcoidosis and some other lymphatic disorders, causing hypercalcaemia and its complications from exposure to sunshine alone or following supplementation. See the discussion regarding vitamin D and sarcoidosis in this recent review [110].

Thus, given the multiple indications of significant health benefits from raising serum 25(OH)D concentrations above 30 or 40 ng/mL as well as the near absence of adverse effects, significant improvements in health at the individual and population levels could be achieved. Methods to achieve optimal health benefits could usefully begin with establishing effect thresholds for different disorders with reasonable certainty while allowing for variations reported with obesity, diabetes, ethnicity, age or gender and by instituting programs to encourage and facilitate raising serum 25(OH)D concentrations through a variety of approaches including sensible solar UVB exposure, vitamin D supplementation and food fortification. A vitamin D fortification program of dairy products initiated in Finland in 2003 eventually resulted in 91% of non-vitamin D supplement users reaching 25(OH)D concentrations >20 ng/mL [111], The rationale and plan for food fortification with vitamin D, which was doubled in 2010, was outlined in 2018 [112].

As for future research, the most efficient way to determine the effects of vitamin D supplementation seems to be to conduct observational studies of individual participants who supplement with vitamin D_3_. A concern regarding such observational studies is that the controls might not be well matched to those supplementing with vitamin D. A way to improve such studies is to use propensity score matching of both groups, as reported in two recent vitamin D studies. One was an examination of the de novo use of vitamin D after the diagnosis of breast cancer [113]. The other was in the study from Spain regarding vitamin D_3_ or calcifediol supplementation and the risk of COVID-19 [88]. Using propensity score matching in observational studies can elevate them to the level of RCTs in terms of examining causality.

### Types of Studies, Strengths and Weaknesses

Many types of studies are used to help determine whether a factor modifies disease risks (incidence, survival, and/or mortality rates). A typical evidence pyramid published in 2018 showed a hierarchy with in vitro studies at the bottom, progressing upward with animal, ecological, cross-sectional, case-control studies, and randomized controlled trials (RCTs) with meta-analyses of RCTs at its apex [114]. Although this pyramid is appropriate for pharmaceutical drugs, it has various limitations when applied to nutrients. Not generally included in such pyramids is an understanding of the mechanisms by which a nutrient or agent of interest works, though that is very important when considering causality for vitamin D in each disorder of interest. For mechanisms regarding vitamin D, the reader is referred to *Vitamin D, 4th Edition* [115] as well as pubmed.gov and scholar.google.com. The main types of studies used for vitamin D are discussed here in the ascending order of classic pyramidal evidence hierarchies. 

Ecological studies consider populations defined geographically and use risk-modifying factors and health outcome population averages; they can be either geographical or temporal. For example, the first indication that better provision of vitamin D reduced cancer risks came from an ecological study of colon cancer mortality rates in the U.S. in relation to annual solar radiation doses [17] and for reduction in cardiovascular disease (CVD) risks from a study showing seasonality in CVD mortality rates [2] and from a similar finding for epidemic influenza in 2006 [116]. The strengths of ecological studies include the inclusion of large numbers of participants, that solar UVB doses have large latitudinal gradients in middle-latitude countries and show large seasonal variations, and that many risk-modifying factors can be used in the analysis as were used for cancer in 2006 in an American ecological study that included alcohol consumption, Hispanic heritage, poverty level, smoking, and urban/rural residence as well as July 1992 solar UVB doses [19]. On the other hand, solar UVB is an important source of vitamin D, but is strongly associated with solar UVA (320–400 nm) radiation which has other health effects such as liberating nitric oxide from subcutaneous nitrogen compounds, which reduces arterial stiffness [117] and thereby reduces blood pressure [118] and the risk of COVID-19 [119]. However, ecological studies performed on post-2000 data generally fail to find inverse correlations between solar UVB and cancer incidence or mortality rates, most likely because people are spending less time in the sun, use more sunblock, are also more likely to be obese and no doubt also because cancer survival rates are now much improved by more effective therapies [120]. Although ecological studies cannot establish causality in isolation, they can provide strong support in combination with other types of studies. 

Cross-sectional studies consider the relationship between many variables and health status at one point in time, revealing associations but unable to establish causality since variables studied may be affected by the disease of interest. Nonetheless, they can provide associational information for comparison with findings from other types of studies; for example, a cross-sectional study of women recently diagnosed with breast cancer in Brazil showed an inverse correlation between serum 25(OH)D concentration and factors used to estimate prognosis and showed an association of low 25(OH)D with increased rates of estrogen receptor-negative tumors [121].

Case-control studies involve measuring variables for those with a particular outcome (cases) with similar individuals without that outcome [122]. The study can be either retrospective, e.g., the history of solar UVB exposure, or contemporaneous with disease incidence, (e.g., serum 25(OH)D concentration). An important strength of case-control studies for cancer and all-cause mortality rate is that they generally provides concomitant serum 25(OH)D concentrations, whereas prospective studies use blood samples from the time of enrollment even though 25(OH)D concentrations change over time, thereby reducing correlations of 25(OH)D with outcomes over time [26]. This effect is especially important for breast cancer which can progress from undetectable to obvious very rapidly and is one of only a few cancers with a pronounced seasonality of incidence [123].

There are several concerns about case-control studies. One is that controls may not be well matched to cases. The way to overcome this concern is to use propensity score matching as conducted in two recent vitamin D studies [88,113]. Another is that the disease itself may affect serum 25(OH)D concentration as has been demonstrated with acute inflammatory diseases (e.g., acute respiratory tract infections [27]) but not for undiagnosed cancers, where inflammation is not generalized. 

Prospective cohort studies generally enroll participants over short periods of time, measure many variables of relevance and draw blood for later analysis, before following participants, commonly over many years before outcome assessment and data analysis using a nested case-control approach. The advantages include the inclusion of many subjects that act as controls against which case risk can be assessed. An important limitation is that variable values can change over time, including serum 25(OH)D concentrations [26]. However, the results of individual cohort studies can be combined for meta-analyses, which often provide the strongest evidence for various health outcomes [124].

An important limitation of observational studies is that most participants have serum 25(OH)D concentrations between 10 and 40 ng/mL [125,126]. Most vitamin D is obtained from solar UVB exposure plus some from animal-based food including meat, fish, eggs [127], and vitamin D-fortified food [112], and supplements. However, the recommended vitamin D supplement value for adults in the U.S. is 600 IU/day up to 70 years of age and 800 IU/day for those over 70 years old [102]. According to changes in serum 25(OH)D with supplementation [12], 600 IU/day can increase 25(OH)D by about 5.6 ng/mL, and 800 IU/day by 7.5 ng/mL.

Thus, most observational studies to date include few participants with 25(OH)D concentrations >40 ng/mL unless they are supplementing with 1000 to 5000 IU/day or more as is the case for the observational studies conducted by GrassrootsHealth.net (accessed on 15 December 2021), as discussed [33,106].

Randomized controlled trials (RCTs) are considered the strongest type of evidence in medical decision making, their strength being that they examine the effect of particular substances and can, therefore, rule out many confounding factors. Unfortunately, vitamin D differs from pharmaceutical drugs in that there are several sources including solar UVB exposure, diet, and supplements, and that serum 25(OH)D concentration–health outcome relationships are non-linear. As a result, vitamin D RCTs generally fail to confirm findings from observational studies [128,129]. Robert Heaney outlined the guidelines for nutrient trials, where the important factor is that nutrient concentration, e.g., 25(OH)D, should drive both trial design and analysis and not the supplemental dosage [47,48]. As a result of using RCT design guidelines evolved for testing pharmaceutical drug efficacy most vitamin D RCTs have failed to find the beneficial effects of supplementation. The primary reasons for such predictable failures include enrolling participants with 25(OH)D concentrations that are relatively high, using relatively low vitamin D doses that cannot raise 25(OH)D values into the normal range, permitting participants (including controls) to take additional supplements, not recognizing that participants may have different vitamin D responses [49] and that there are different 25(OH)D thresholds for different health benefits.

Although not on the standard evidence pyramid, Mendelian randomization (MR) studies are also suggested to be valuable for establishing causality for vitamin D for various health outcomes. MR studies compare the estimated effect of SNPs associated with variation in 25(OH)D concentrations on the health outcomes seen in large numbers of participants, often up to 100,000. Although some MR studies report inverse correlations between the SNPs increasing serum 25(OH)D and several health outcomes [130] such as the incidence of multiple sclerosis [131] and ovarian cancer [132], no such effects were seen for eight other types of cancer [133]. The primary reasons for MRA failure are likely to include the fact that total SNP-induced variation in 25(OH)D has often been less than 25(OH)D assay variance [134] and that genome-wide association studies’ (GWAS) analyses of the total percentage of SNP effects are made on the 25(OH)D data as a whole, although such data is non-linear with much of it lying in the low and high plateaus of the 25(OH)D–health outcome relationships, a problem that the GWAS analysis of 25(OH)D data stratified for different ranges of 25(OH)D efficacy might overcome [135]. That this is the case for mortality rates was shown in two recent articles, one [10] where GWAS serum 25(OH)D concentration was stratified at <10 ng/mL, 10–20 ng/mL, 20–30 ng/mL, and >30 ng/mL and significantly increased risk was only present at 25(OH)D <10 ng/mL for all-cause mortality, cardiovascular mortality, and non-CVD and non-cancer mortality with trends for increases in risk for stroke and cancer mortality. The other [12], using genetic increases in serum 25(OH)D calculated for 100 equal strata of measured serum 25(OH)D showed similar results for CVD with risk reduction for increases in 25(OH)D values up to ~20 ng/mL. 

## Figures and Tables

**Table 1:** Mortality rates (deaths/100,000/year) as well as obesity rates in 2016.

Outcome	Germany M	Germany F	Japan M	Japan F	Saudi Arabia M	Saudi Arabia F	USA M	USA F
All causes	504	328	401	217	777	608	592	404
CVD	160	106	93	54	329	261	167	104
IHD	95	54	42	22	219	154	106	56
Stroke	24	20	33	20	86	76	24	21
Cancer	148	97	139	76	64	57	132	99
Breast	0.2	19	0.1	10	0	9	0.2	18
Lung	36	17	33	10	7	3	33	23
COPD	26	15	19	7	15	12	35	28
Lower respiratory tract disease	12	7	36	17	46	42	13	10
Diabetes mellitus	12	8	5	2	30	25	19	12
Alcohol abuse	7	2	0.5	0.1	0.5	0.1	4	1
Alzheimer’s disease	15	16	6	5	46	44	28	35
Obesity (%) – 2016	22		4		35		36	

Table 1. Global Health Estimates 2016: Deaths by Cause, Age, Sex, by Country and by Region, 2000–2016. Geneva, World Health Organization; 2018. Obesity data from https://obesity.procon.org/global-obesity-levels/ (accessed on 15 December 2021). COPD, chronic obstructive pulmonary disease; CVD, cardiovascular disease; F, female; IHD, ischemic heart disease; M, male.

**Table 2:** Meta-analyses of observational studies of individual cancer site risks in relation to serum 25(OH)D concentrations #.

Cancer Site	*N* Studies	Type of Study	RR (95% CI) (High vs. Low)	Reference
Bladder	5	Cc	0.70 (0.56 to 0.88)	[28]
Bladder	2	Cohort	0.80 (0.67 to 0.94)	[28]
Breast	44	Cc	0.57 (0.48 to 0.66)	[29]
Breast	6	Cohort	1.17 (0.92 to 1.48)	[29]
Colorectal	11	Cc	0.60 (0.53 to 0.68) *	[30]
Colorectal	6	Cohort	0.80 (0.66 to 0.97) *	[30]

(*) fixed effects model; # https://pubmed.ncbi.nlm.nih.gov/ (accessed on 15 December 2021).

**Table 3:** Meta-analyses of breast cancer risk from vitamin D RCTs.

Cancer Site	*N* Studies	Outcome	RR (95% CI)	Reference
Breast	9	Incidence	0.96 (0.86 to 1.07)	[38]
Breast	5	Mortality	0.87 (0.79 to 0.96)	[38]

**Table 4:** Meta-analyses of cancer incidence and mortality rates from observational studies.

Cancer Site	*N* Studies	Outcome	Low, High 25(OH)D (ng/mL)	RR Low 25(OH)D	RR High 25(OH)D	Ratio High to Low	Reference
Total	8	Incidence	1, 21	1.31 (95% CI, 0.87 to 2.05)	0.71 (95% CI, 0.55 to 0.92)	0.54	[39]
	17	Mortality	10, 40	1.47 (95% CI, 1.11 to 1.88)	0.87 (95% CI, 0.75 to 1.02)	0.59	[39]

**Table 5:** Optimal 25(OH)D concentrations for various health outcomes.

Outcome	Type of Evidence	Optimal 25OHD	Reference
All-cause mortality rate	Observational study of 25(OH)D concentration due to vitamin D supplementation	>30 ng/mL	[8]
Alzheimer’s disease and dementia	Meta-analysis of observational studies	>25 ng/ml	[93]
Breast cancer	Observational study of 25(OH)D concentration due to vitamin D supplementation	>60 ng/mL	[33]
Colorectal cancer	Meta-analysis of observational studies	30–40 ng/mL	[34]
Cardiovascular disease	Observational study of the CVD mortality rate for CVD patients	>30 ng/mL	[9]
Myocardial infarction	Observational study of 25(OH)D concentration due to vitamin D supplementation‘1n	>30 ng/mL	[8]
SARS-CoV-2 infection	Retrospective observational study	>50 ng/mL	[75]
COVID-19 mortality	Retrospective cohort study	>60 ng/mL	[82]
Diabetes mellitus type 2	RCT with an analysis of intratrial 25(OH)D for prediabetes patients	>50 ng/mL	[70]
Gene expression	Clinical trial	>40 ng/mL	[45]
Hypertension	Observational study of 25(OH)D concentration due to vitamin D supplementation	>40 ng/mL	[16]
Preterm delivery	Observational study of 25(OH)D concentration due to vitamin D supplementation	>40 ng/mL	[106]
